# Epidemiology and Genetic Characterization of *Leishmania* RNA Virus in *Leishmania* (*Viannia*) spp. Isolates from Cutaneous Leishmaniasis Endemic Areas in Panama

**DOI:** 10.3390/microorganisms12071317

**Published:** 2024-06-27

**Authors:** Armando Assair Bonilla, Vanessa Pineda, José Eduardo Calzada, Azael Saldaña, Marcia Dalastra Laurenti, Stephanie Goya, Leyda Abrego, Kadir González

**Affiliations:** 1 Programa de Maestría en Ciencias Parasitológicas, Facultad de Ciencias Naturales, Exactas y Tecnología, Universidad de Panamá, Panama 3366, Panama; aabonillaf22@gmail.com; 2 Departamento de Investigación en Parasitología, Instituto de Conmemorativo Gorgas de Estudios de la Salud, Panama 0816-02593, Panama; vpineda@gorgas.gob.pa (V.P.); jcalzada@gorgas.gob.pa (J.E.C.); 3 Facultad de Medicina Veterinaria, Universidad de Panamá, Panama 3366, Panama; 4 Centro de Investigación y Diagnóstico de Enfermedades Parasitarias (CIDEP), Facultad de Medicina, Universidad de Panamá, Panama 3366, Panama; azael.saldana@up.ac.pa; 5 Laboratório de Patologia de Moléstias Infecciosas, Faculdade de Medicina, Universidade de São Paulo, São Paulo 05508-270, SP, Brazil; mdlauren@usp.br; 6 Department of Laboratory Medicine and Pathology, University of Washington, Seattle, WA 98195, USA; goyastephanie@gmail.com; 7 Departamento de Investigación en Virología y Biotecnología, Instituto Conmemorativo Gorgas de Estudios de la Salud, Panama 0816-02593, Panama; 8 Departamento de Microbiología y Parasitología, Facultad de Ciencias Naturales, Exactas y Tecnología, Universidad de Panamá, Panama 3366, Panama; 9 Departamento de Microbiología Humana, Facultad de Medicina, Universidad de Panamá, Panama 3366, Panama

**Keywords:** LRV, Leishmaniavirus, *Leishmania Viannia*, leishmaniasis, phylogenetic, sequencing, Panama

## Abstract

*Leishmania* (*Viannia*) spp. can harbor a double-stranded RNA virus known as Leishmania RNA virus 1 (LRV-1), whose presence has been reported in nine countries across the Americas and seven *Leishmania* species. Here, we studied 100 *Leishmania* (*Viannia*) isolates from patients with cutaneous leishmaniasis collected from different endemic areas in Panama from 2016 to 2022. We identified *L.* (*V.*) *panamensis*, *L.* (*V.*) *guyanensis*, *L.* (*V.*) *braziliensis/guyanensis* hybrid, and *L.* (*V.*) *panamensis* sp.1. (genetic variant). LRV-1 was detected by RT-PCR in 9% of *L. (Viannia)* isolates (eight cases in *L.* (*V.*) *panamensis*, and one in *L.* (*V.*) *guyanensis)*. Phylogenetic analysis based on sequencing data classified all LRV-1 isolates within genotype A, suggesting that LRV phylogenetic proximity is closely aligned with geographical distribution or to the phylogenetic proximity of the *Leishmania* host in the case of the *L.* (*V.*) *panamensis* and *L.* (*V.*) *guyanensis* in Panama.

## 1. Introduction

*Leishmania* spp. can be infected by a nonenveloped double-stranded RNA virus of the genus Leishmaniavirus (LRV, family Totiviridae). One species, LRV-1, inhabits species of the subgenus *Viannia*, while the other, LRV-2, is found in the representatives of the species of the subgenus *Leishmania* and *Sauroleishmania* [1,2,3,4,5,6,7,8,9,10]. Since the first report of LRV in an isolate of *L.* (*V.*) *guyanensis* [11], researchers have tried to understand the importance of this virus and its relationship with *Leishmania* [5,12,13,14]. Several studies have associated the presence of LRV with the exacerbation of leishmaniasis, including transformation to mucocutaneous form and treatment failure [14,15,16,17].

The viral genome ranges in size from 4.9 to 5.3 kb and encodes the capsid protein (ORF2) and the RNA-dependent RNA polymerase (ORF3) [18]. It also contains the so-called ORF1 located at the 5′ end that does not encode a protein but it presumably plays a role in RNA stability and translation [19].

In the Americas, LRV-1 presence has been described in French Guiana, Suriname, Brazil, Peru, Bolivia, Ecuador, Colombia, Costa Rica, and Panama [8,17,20,21,22,23,24]. Additionally, LRV-2 has been reported in *L. (L.) infantum* isolates from both canine and human host in Brazil [25]. LRV-1 has been detected in several *Leishmania* species such as *L.* (*V.*) *guyanensis*, *L.* (*V.*) *braziliensis*, *L.* (*V.*) *panamensis*, *L.* (*V.*) *lainsoni*, *L.* (*V.*) *shawi*, *L.* (*V.*) *peruviana* and *L.* (*V.*) *naiffi* [2,5,7,9,16,18,26,27,28,29,30]. Recent phylogenetic studies have classified LRV-1 into eight genotypes (A-H) according to the *Leishmania* species they infect [13,18]. According to this classification, LRV-1 found in *L.* (*V.*) *guyanensis* can be categorized into genotypes A-E, *L.* (*V.*) *braziliensis* as F, *L.* (*V.*) *shawi* as G, and *L.* (*V.*) *naiffi* as H [18,31].

Panama is considered an endemic country for cutaneous leishmaniasis (CL) with an average of 1000 new cases annually, predominantly caused by *L.* (*V.*) *panamensis* [32,33]. According to the Pan American Health Organization (PAHO) report in 2023, there has been a notable increase in the risk of transmission in recent years, elevating the risk classification from moderate to high [33]. Previously, we have confirmed the presence of LRV-1 in 11 *L.* (*V.*) *panamensis* isolates from CL endemic regions in Panama [34]. However, a comprehensive analysis to characterize the virus at the genotype level remains pending. Furthermore, the evaluation of LRV presence in other *Leishmania* species circulating in Panama remains unexplored, even though the circulation of *L.* (*V.*) *guyanensis*, *L.* (*V.*) *braziliensis*, *L.* (*V.*) *naiffi*, *L. (V.). braziliensis/guyanensis* hybrids, and a genetic variant of *L.* (*V.*) *panamensis* named sp.1 have been recorded in the country [34,35,36,37]. In this regard, LRV-1 has been already described in *L.* (*V.*) *guyanensis*, *L.* (*V.*) *naiffi*, and *L.* (*V.*) *braziliensis* in various South American countries [17,20,22,23].

In this work, we present a retrospective study of LRV-1 in clinical isolates of *Leishmania* (*Viannia*) collected in Panama. We demonstrate that Panamanian LRV-1 circulates in *L.* (*V.*) *panamensis* and *L.* (*V.*) *guyanensis* and belongs to the genotype A.

## 2. Materials and Methods

### 2.1. Study Design and Leishmania Isolates

This is a cross-sectional study with 100 isolates of *Leishmania* (*Viannia*) spp. sampled from skin lesions of patients who attended the Unidad de Diagnóstico, Investigación Clínica y Medicina Tropical of the Instituto Conmemorativo Gorgas de Estudios de la Salud (ICGES), Panama, between 2016 and 2022 (Table S1). These isolates were previously identified using the RFLP/PCR-Hsp70 method and were cryopreserved in the ICGES biobank [38]. Additionally, the amplified products of the Hsp70 gene for the LRV-1-positive *Leishmania* isolates were sequenced to confirm the species by reconstructing a Neighbor-Joining phylogenetic tree in MEGA X software using the Kimura 2-parameter model and branch support assessed with 2000 bootstrap replicates [37,38]. Nucleotide sequences of the LRV-1-positive *Leishmania* isolates are available in NCBI GenBank under the accession numbers PP796356 to PP796364.

### 2.2. Ethical Considerations

This study and the retrospective use of biological samples were carried out with the approval of the ICGES Institutional Bioethics Committee (code 267/CBI/ICGES/21). The study did not involve direct work with human subjects or animals.

### 2.3. Preparation of Isolates

Parasites from the biobank were thawed and grown in Schneider medium enriched with 25% fetal bovine serum (FBS). Once the stationary phase was reached, RNA was obtained from the parasite pellet using the QIAGEN RNeasy Mini kit (QIAGEN, Redwood City, CA, USA) according to the manufacturer’s instructions. The extracted RNA was quantified using NanoDrop 2000 equipment (Thermo Fisher Scientific, Wilmington, DE, USA) and stored at −80 °C until use.

### 2.4. RT-PCR for LRV-1 Detection

The SuperScript IV First-Strand Synthesis SuperMix kit was used for the cDNA re-verse transcription according to the manufacturer’s instructions. Each sample was analyzed by PCRs using four different sets of primers: (1) fragment of 240nt from ORF1 (LRV-1F: 5′-ATGCCTAAGAGTTTGGATTCG-3′ and LRV-1R: 5′-ACAACCAGACGATTGCTGTG-3′) [31]; (2) fragment of 850nt spanning from end of ORF1 to the beginning of the ORF2 (LRV-1ORF1: 5′-ATGCCTAAGAGTTTGGATTCG-3′ and LRV-1ORF2: 5′-AATCAATTTTCCCAGTCATGC-3′) [7], (3) fragment of 488nt spanning the capsid gene (UNIVF: 5′-TWGCRCACAGTGAYGAAGG-3′ and UNIVR: 5′-CWACCCARWACCABGGBGCCAT-3′) [9,39], and (4) fragment of 942nt within the ORF3 (LRV3sF: 5′-ATGCATGTHGGTGATGACATHYTRATGTC-3′, LRV4asR: 5′-TGAGCCATTGARGTYGCTTCRTTRTAYGGA-3′ and 5LRVs1F: 5′-ATCATGGCCCAGGCYAGCTGA-3′) [13]. The PCR products were verified on 1% agarose gel containing ethidium bromide. Three reference strains were used as controls: *L.* (*V.*) *guyanensis*/LRV-1-positive control (MHOM/BR/1975/WR4147), *L.* (*V.*) *panamensis*/LRV-1-negative control (MHOM/PA/1971/LS94), *L.* (*V.*) *braziliensis*/LRV-1-negative control (MHOM/BR/1975/M2903).

### 2.5. Purification and Sequencing

The amplified products were purified using ExoSAP-IT Express PCR Product Cleanup, and the Sanger sequencing reaction was performed using the BigDye Terminator v3.1 cycling kit using the primers previously described [13,39]. Sanger reaction products were purified with the BigDye XTerminator kit (Applied Biosystems, Waltham, MA, USA) and analyzed using the 3130xl Genetic Analyzer sequencer (Applied Biosystems, Foster, CA, USA). Nucleotide sequences were confirmed using Sequencher version 5.4.6 DNA sequence analysis software and aligned using the BioEdit v7.0.9.0 program.

### 2.6. Construction of Phylogenetic Tree

Nucleotide sequences were aligned using MAFFT v7.490 [40] and visualized with Aliview v1.28 [41]. The nucleotide substitution model for each dataset was selected using ModelFinder implemented within IQ-TREE v2.2.0 [42]. Bayesian phylogenetic trees were constructed using MrBayes v3.2.7 with two parallel runs of four Monte Carlo Markov Chains (MCMC) with 10 million generations sampled every 10,000 generations, resulting in a total of 1000 trees [43]. The MCMC convergence was evaluated in TRACER v.1.5 with an effective sample size (ESS) > 200; the initial 10% of the run length was discarded as burn-in. The consensus tree was summarized from sampled trees and visualized with FigTree v.1.4.4. Clades with posterior probability <0.6 were collapsed. Nucleotide sequences are available in NCBI GenBank under the accession numbers listed in Table 1.

## 3. Results

### 3.1. Geographic Distribution of L. (Viannia) Species and LRV-1 in Panama

**Figure 1:**
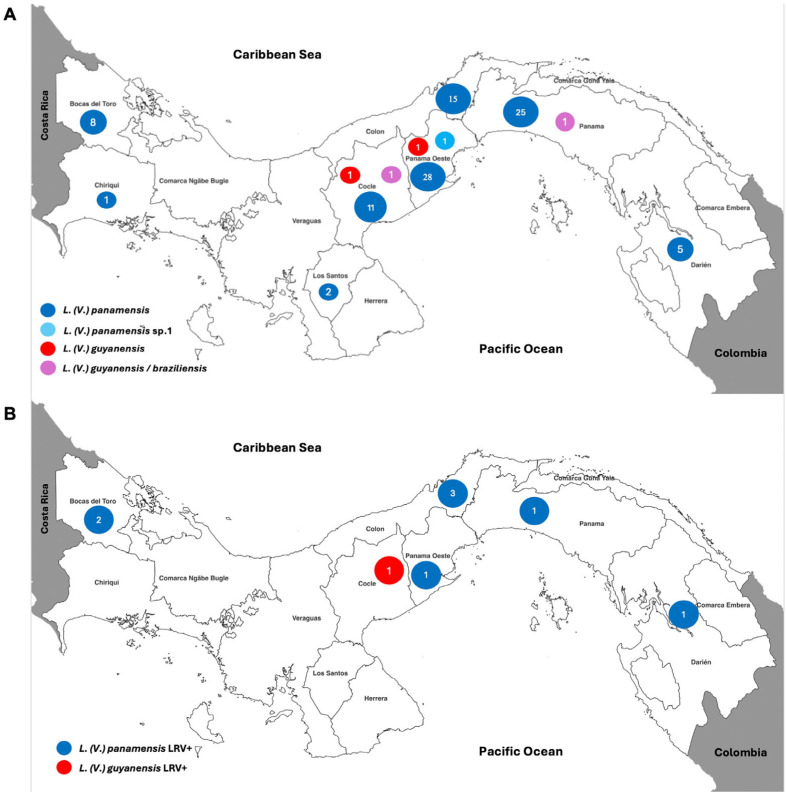
Distribution of *Leishmania* (*Viannia*) isolates analyzed in this study. (**A**) All isolates (n = 100). (**B**) Virus-positive isolates (n = 9). The colors show the *Leishmania* species, and the number inside the circle indicates the number of isolates analyzed.

We analyzed the presence of LRV-1 in 100 *Leishmania* (*Viannia*) isolates from confirmed cutaneous leishmaniasis (CL) cases in Panama (Figure 1A). *Leishmania* (*V.*) *panamensis* predominated with 95 isolates, followed by 2 of *L.* (*V.*) *guyanensis*, 2 of *L.* (*V.*) *guyanensis/braziliensis* hybrids, and 1 of *L.* (*V.*) *panamensis* genetic variant sp.1. The *L.* (*V.*) *panamensis* isolates were distributed widespread throughout the country, while the other species were concentrated in the provinces of Panama, Panama Oeste, and Cocle, geographically close to the Panama Canal (Figure 1A).

Nine isolates were positive for LRV-1 (9/100: 9%), of which eight were found in *L.* (*V.*) *panamensis* and one in *L.* (*V.*) *guyanensis* (Figure S1). All positive isolates corresponded to male patients. The age of the patients ranged between 27 and 62 years, with a mean of 42 years. Most of the patients had single lesions (7/9: 77.78%), with a range of 1 to 11 lesions per patient. The mean of the development time of the lesion was 42 days, with a range of 30–60 days. The lesions were mainly distributed on the arms (4/9, 44.44%) and legs (4/9, 44.44%). Only one patient presented with 11 ulcerated lesions: 7 on the face, 1 on the leg, 1 on the arm, and 2 on the back. Of the 91 LRV-1-negative isolates, the majority were men (65%). The age of the patients ranged between 1 and 75 years, with a mean of 31 years. Most of the patients had single lesions (81/91: 89.01%), with a range of 1 to 3 lesions per patient. The mean of the development time of the lesion was 35 days, with a range of 15–120 days. The lesions were mainly distributed on the arms (72/91, 79.12%), legs (17/91, 18.68%), back (1/91, 1.1%), and face (1/91, 1.1%).

Three of the LRV-1-positive *L.* (*V.*) *panamensis* isolates originated from Colon, two from Bocas del Toro, and three from Panama Oeste, Panama, and Darien. Additionally, one isolate of *L.* (*V.*) *guyanensis* was obtained from Cocle (Figure 1B).

Assessment of disease severity concerning LRV-1 presence demonstrated that two *L.* (*V.*) *panamensis* isolates were associated with complicated CL cases: one with 11 lesions, and one with treatment failure despite undergoing three complete rounds of pentavalent antimonial treatment (Table 1). In contrast, no complicated CL cases were observed in LRV-1-negative isolates. The association of complicated CL cases with LRV presence (2/9: 22.22% vs. 0/91: 0%) was statistically significant according to the N-1 chi-square test for (*p* < 0.0001) [44].

### 3.2. Phylogenetic Analysis of LRV-1 Detected in Panama

Our sequencing efforts allowed us to characterize a 240nt fragment of the ORF1 of all LRV-1 from the *L.* (*V.*) *panamensis* isolates. For the *L.* (*V.*) *guyanensis* isolate, we were able to reconstruct a partial genome including 790nt of ORF1 and the beginning of ORF2, 678nt within ORF2, and 942nt of ORF3 [13,29,39].

**Figure 2:**
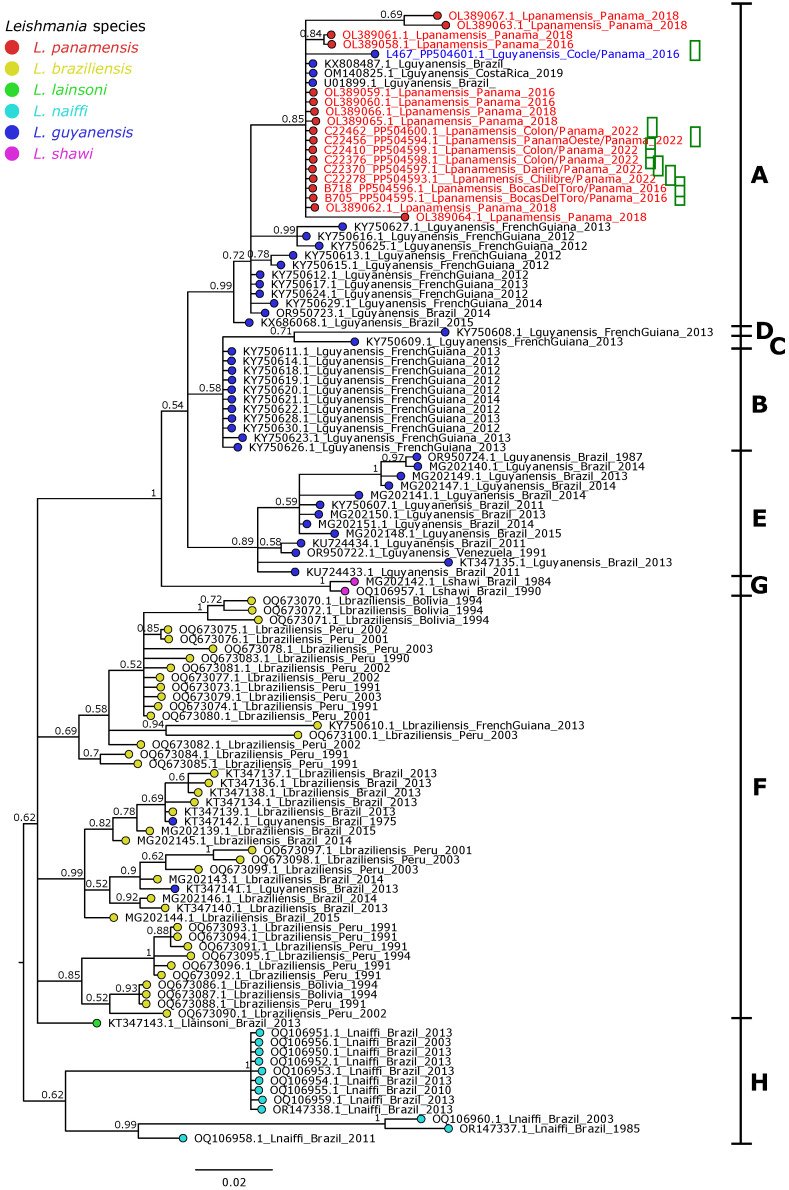
Bayesian phylogenetic tree partial ORF1 of the LRV-1. The phylogenetic tree was constructed from an alignment spanning approximately 240nt of the ORF1. Colored dots next to the sequence name indicate the *Leishmania* (*Viannia*) species. Sequences collected in Panama are highlighted in red for LRV-1 isolated from *L.* (*V.*) *panamensis* and in blue for LRV-1 isolated from *L.* (*V.*) *guyanensis*. Sequences obtained in this study are marked with green arrows and named by strain ID, accession number, host species, geographic region, and year of collection. Sequences from NCBI GenBank are identified by accession number, host species, country, and year of collection. Bars at the right of the phylogenetic clades denote the genotypes. Posterior probability >0.6 is shown at clade nodes.

The Bayesian phylogenetic tree for the 240nt ORF1 fragment, including the same region from all LRV-1 sequences available in the NCBI GenBank database (up to March 2024), revealed a well-defined clustering pattern associated with the *Leishmania* host. Notably, all LRV-1 identified in Panama consistently clustered within genotype A, regardless of whether they infected *L.* (*V.*) *panamensis* or *L.* (*V.*) *guyanensis* (Figure 2). It is noteworthy that all LRV-1 sequences detected in Panama between 2016 and 2022 (from this study and the previous one) exhibited close evolutionary relationships with the sequence of *L.* (*V.*) *guyanensis* collected in Costa Rica in 2019 (OM140825) and two sequences from the Northeast of Brazil (KX808487 and U01899). Additional analysis of the *L.* (*V.*) *guyanensis* partial genome confirmed its association with the sequences of genotype A (Figure S2).

## 4. Discussion

Cutaneous leishmaniasis is a public health problem of major concern in Panama, predominantly caused by *L.* (*V.*) *panamensis* [32,33]. Since the discovery of the LRV-1 infecting different *Leishmania* species, several studies have tried to unravel the epidemiological importance of this virus and its relationship with *Leishmania* spp. [11,17,21,22,24,34,45,46].

Here, we present a surveillance study of *Leishmania* and LRV-1 in Panama from 2016 to 2022 to ascertain the LRV-1 positivity rate, genotypic classification, and host range. We studied 100 *Leishmania* (*Viannia*) isolates and found a 9% LRV-1 positivity rate, which is lower than the previous report in Panama (20%) [35]. However, the N-1 chi-square test does not confirm significance of this difference (*p* = 0.0582). Apparently, the viruses have mosaic distribution in the populations of leishmaniae, and therefore, different studies have reported a wide range of virus detection rates—from 2% to 70% [17,21,24,47,48]. Another reason may be the loss of the virus upon cultivation, leading to the underestimation of the virus infection rate. Analysis of parasites directly from the lesion is devoid of such a potential drawback [45].

Our analysis revealed the predominance of *L.* (*V.*) *panamensis* among leishmaniae in Panama but also demonstrated the presence of other species and genetic variants in provinces adjacent to the Panama Canal. However, LRV-1 was equally distributed throughout the country and was documented in Bocas del Toro alongside the previously documented Panama Oeste, Panama, Cocle, Colon, and Darien [34]. It was not possible to detect the virus in the few cases of *L.* (*V.*) *guyanensis/braziliensis* hybrids and *L.* (*V.*) *panamensis* genetic variant sp.1. Notably, we documented for the first time LRV-1 infecting *L.* (*V.*) *guyanensis* isolates in the Cocle region.

The Panama isthmus is the geographical link between Central and South America, providing a biological corridor between Colombia and Costa Rica, allowing the entry to Panama of the LRV-infected *Leishmania* spp., in vectors and reservoirs [38]. Another point to consider is that Panama currently serves as a significant transit point for migrants traveling through South America on their northward journey toward the USA or Canada. This transit poses a risk for the introduction of new *Leishmania* spp. harboring the LRV-1, potentially facilitating the establishment of these parasites and the virus they carry within Panama’s natural transmission cycle [37,49].

Overall, our findings aligned with previous studies confirming the infection of LRV-1 in *L.* (*V.*) *guyanensis* and *L.* (*V.*) *panamensis* [7,20,34,45,50,51,52]. Given that this species is linked to a high incidence of CL cases in Panama, it provided an ideal context for analyzing a representative number of parasite isolates and identifying and characterizing the LRVs present. According to the phylogenetic classification proposed by Tirera et al. [13], all our LRV-1 isolates, whether from *L.* (*V.*) *panamensis* isolate or *L.* (*V.*) *guyanensis*, are classified within genotype A [13,18]. This finding is significant given the close phylogenetic proximity between *L.* (*V.*) *panamensis* and *L.* (*V.*) *guyanensis*, which explains the presence of evolutionarily similar LRV-1 in isolates of both species [20,36,53,54,55,56].

Our analysis further revealed that LRV-1 sequences from Panama were evolutionarily close to those from Costa Rica and Northeast of Brazil, supporting previous hypotheses that the phylogenetic proximity of viral isolates aligns more closely with the geographical distribution of the *Leishmania* host [46]. Additionally, there is a possibility that LRV-1 can infect *L.* (*V.*) *guyanensis* or *L.* (*V.*) *panamensis* indiscriminately, unlike what has been observed in other species such as *L.* (*V.*) *braziliensis*, *L.* (*V.*) *shawi*, or *L.* (*V.*) *naiffi* [18]. In other words, the phylogenetic analysis of the LRV-1 partial genome from *L.* (*V.*) *guyanensis* in Panama reaffirmed its association with genotype A (Figure S2) [13,18].

LRV-1 is an important component in the dynamics of infection by parasites of the genus *Leishmania*, influencing the virulence of the parasite and the immune response of the vertebrate host [7,14,17,26,57,58,59]. In this context, the analysis of clinical characteristics among the LRV-1-positive cases showed that most of our evaluated patients with CL were classified as non-serious according to criteria established by the Infectious Diseases Society of America (IDSA). However, in the panel of evaluated parasites, we encountered two isolates of *L.* (*V.*) *panamensis* LRV+ associated with patients experiencing clinical complications: one with 11 lesions, while another exhibited treatment failure despite undergoing three cycles of pentavalent antimonial therapy for a single lesion. These findings are consistent with previous reports suggesting that, unlike in other species, the presence of LRV-1 in *L.* (*V.*) *panamensis* does not necessarily exacerbate clinical manifestations as observed in *L.* (*V.*) *guyanensis* and *L.* (*V.*) *braziliensis* [26,34,60]. Likewise, our study did not observe an association between the presence of LRV-1 and complicated forms of CL in the single *L.* (*V.*) *guyanensis* LRV-1+ isolate analyzed. Although some authors have reported that LRV presence does not invariably lead to treatment failures or clinical complications, it is important to emphasize that the incidence of CL caused by *L.* (*V.*) *guyanensis* is markedly low in Panama compared to other endemic areas of South America [15,17,21]. The study has limitations, including the small number of LRV-1-positive cases (although we analyzed a total of 100 *L. (Viannia*) isolates) and the inability to obtain viral complete genomes for all the isolates. Nevertheless, our study provides insights into the evolution and geographical distribution of LRV-1 and the role of coevolution with the *Leishmania* (*Viannia*) species.

## 5. Conclusions

Our study confirms the presence of LRV-1 in both *L.* (*V.*) *panamensis* and *L.* (*V.*) *guyanensis* isolates from CL endemic areas in Panama, with phylogenetic analysis classifying them within the genotype A. Sharing of the same viral lineage suggests that these two *Leishmania* spp. can exchange their viruses, which is apparently facilitated by the close relationship of both parasites. While most LRV-1+ isolates were associated with uncomplicated CL cases, we identified two cases of *L.* (*V.*) *panamensis*/LRV+ isolates linked to clinical complications and treatment failure. However, the association between LRV-1 presence and disease severity or treatment outcomes in Panama remains inconclusive. Further investigation is needed to clarify the impact of LRV-1 on the pathogenesis of *Leishmania* (*Viannia*) spp. infections.

## Figures and Tables

**Table 1:** Description of the *Leishmania* (*Viannia*) isolates with LRV-1.

Isolate ID	Species	Geographic Region	Year of Collection	Number of Lesions	GenBank Accession Number
L467	*L.* (*V.*) *guyanensis*	Cocle	2016	1	PP504601
B705	*L.* (*V.*) *panamensis*	Bocas del Toro	2016	1	PP504595
B718	*L.* (*V.*) *panamensis*	Bocas del Toro	2016	1	PP504596
C22-278	*L.* (*V.*) *panamensis*	Panama	2022	11	PP504593
C22-370	*L.* (*V.*) *panamensis*	Darien	2022	1	PP504597
C22-376	*L.* (*V.*) *panamensis*	Colon	2022	4	PP504598
C22-410	*L.* (*V.*) *panamensis*	Colon	2022	1 *	PP504599
C22-462	*L.* (*V.*) *panamensis*	Colon	2022	1	PP504600
C22-456	*L.* (*V.*) *panamensis*	Panama Oeste	2022	1	PP504594

* *Leishmania* LRV-positive isolated from a patient with three cycles of pentavalent antimonial.

## Data Availability

The sequences used to support the findings of this study are available from GenBank with the accession numbers: PP504593-PP504601 and PP796356-PP796364.

## Supplementary Materials

The following supporting information can be downloaded at: https://www.mdpi.com/article/10.3390/microorganisms12071317/s1, Table S1: General description of the *Leishmania* (*Viannia*) spp. isolates analyzed for the detection of LRV-1. Figure S1. Neighbor-Joining phylogenetic tree of the hsp70 sequences from *Leishmania* (*Viannia*) constructed with MEGAX software. Sequences from this study are highlighted in yellow and named, including the sample ID, the GenBank accession number, the *Leishmania* species, and the sequence ID. Reference sequences’ names include the GenBank accession number, the *Leishmania* species, and the sequence ID. Bootstrap values above 50% (2000 replicates) are shown above for the node branches. All gapped positions were removed for each sequence pair (pairwise deletion option). Figure S2. Bayesian phylogenetic tree of LRV-1 genomes. It was inferred with the concatenated sequences of four genomic fragments representing all three ORFs of the LRV-1 isolate from *L.* (*V.*) *guyanensis* in Panama (highlighted in blue) and reference genomes of all genotypes of LRV-1 in *L.* (*V.*) *guyanensis* (LVRg). Genotype F (LRV-1 in *L.* (*V.*) *braziliensis*, LRVb) is selected as an outgroup to root the tree. Posterior probability >0.6 is reported at clade nodes.
