# Supplementary material for: Epidemiology and Genetic Characterization of Leishmania RNA Virus in Leishmania (Viannia) spp. Isolates from Cutaneous Leishmaniasis Endemic Areas in Panama
Source: Microorganisms. 2024 Jun 27;12(7):1317. doi: 10.3390/microorganisms12071317 (PMC11279101; doi:10.3390/microorganisms12071317)
Supplement: Supplementary file 1 [file microorganisms-12-01317-s001.zip › Suplementary Table S1.pdf]

Table S1: General description of the *Leishmania (Viannia)* spp. isolates analyzed for the detection of LRV-1.

No.	Identification	Year of isolation	Region	Species
1	L16-34	2016	Panamá	*L. (V.) panamensis*
2	L18-151	2018	Panamá Oeste	*L. (V.) panamensis*
3	L18-163	2018	Darién	*L. (V.) panamensis*
4	L18-167	2018	Panamá Oeste	*L. (V.) panamensis*
5	L18-185	2018	Coclé	*L. (V.) panamensis*
6	L18-186	2018	Colón	*L. (V.) panamensis*
7	L18-288	2018	Panamá	*L. (V.) panamensis*
8	L18-299	2018	Panamá	*L. (V.) panamensis*
9	L18-305	2018	Panamá	*L. (V.) panamensis*
10	L18-306	2018	Panamá Oeste	*L. (V.) panamensis*
11	L18-420	2018	Panamá	*L. (V.) panamensis*
12	L19-123	2019	Coclé	*L. (V.) panamensis*
13	L19-148	2019	Panamá	*L. (V.) panamensis*
14	L19-150	2019	Panamá	*L. (V.) panamensis*
15	L19-160	2019	Los Santos	*L. (V.) panamensis*
16	L19-169	2019	Panamá Oeste	*L. (V.) panamensis*
17	L19-171	2019	Colón	*L. (V.) panamensis*
18	L19-18	2019	Panamá	*L. (V.) panamensis*
19	L19-180	2019	Panamá Oeste	*L. (V.) panamensis*
20	L19-187	2019	Los Santos	*L. (V.) panamensis*
21	L19-25	2019	Panamá Oeste	*L. (V.) panamensis*
22	L19-274	2019	Panamá	*L. (V.) panamensis*
23	L19-284	2019	Panamá Oeste	*L. (V.) panamensis*
24	L19-324	2019	Panama	*L. (V.) panamensis*
25	L19-40	2019	Panamá Oeste	*L. (V.) panamensis*
26	L19-41	2019	Darién	*L. (V.) panamensis*
27	L19-44	2019	Panamá Oeste	*L. (V.) panamensis*
28	L19-55	2019	Panamá Oeste	*L. (V.) panamensis*
29	L19-56	2019	Chiriquí	*L. (V.) panamensis*
30	L19-65	2019	Panamá Oeste	*L. (V.) panamensis*
31	L19-76	2019	Panamá Oeste	*L. (V.) panamensis*
32	L19-82	2019	Panamá Oeste	*L. (V.) panamensis*
33	L19-85	2019	Panamá, Panamá	*L. (V.) panamensis*
34	L20-22	2020	Colón	*L. (V.) panamensis*
35	L20-26	2020	Panamá	*L. (V.) panamensis*
36	L20-28	2020	Colón	*L. (V.) panamensis*
37	L20-38	2020	Panamá Este	*L. (V.) panamensis*
38	L20-47	2020	Panamá Oeste	*L. (V.) panamensis*

39	L20-73	2020	Panamá, Panamá	*L. (V.) panamensis*
40	L20-74	2020	Panamá, Panamá	*L. (V.) panamensis*
41	L20-85	2020	Colón	*L. (V.) panamensis*
42	L21-03	2021	Panamá	*L. (V.) panamensis*
43	L21-05	2021	Las Mañanitas	*L. (V.) panamensis*
44	L21-09	2021	Colón	*L. (V.) panamensis*
45	L21-21	2021	Panamá Oeste	*L. (V.) panamensis*
46	L21-22B	2021	Panamá Oeste	*L. (V.) panamensis*
47	L21-25	2021	Panamá Este	*L. (V.) panamensis*
48	L21-29	2021	Panamá	*L. (V.) panamensis*
49	L21-34	2021	Coclé	*L. (V.) panamensis*
50	L21-36	2021	Panamá Oeste	*L. (V.) panamensis*
51	L21-38	2021	Panamá	*L. (V.) panamensis*
52	L21-39	2021	Panamá Oeste	*L. (V.) panamensis*
53	L410	2015	Coclé	*L. (V.) guyanensis/ braziliensis*
54	L72	2015	Panamá	*L. (V.) guyanensis/ braziliensis*
55	L467	2015	Coclé	*L. (V.) guyanensis*
56	L119	2016	Panamá Oeste	*L. (V. guyanensis*
57	L22-211	2022	Panamá	*L. (V.) panamensis*
58	C22-370	2022	Darién	*L. (V.) panamensis*
59	C22-376	2022	Colón	*L. (V.) panamensis*
60	L706	2016	Bocas del Toro	*L. (V.) panamensis*
61	L22-417	2022	Darién	*L. (V.) panamensis*
62	C22-278	2022	Panamá	*L. (V.) panamensis*
63	C22-410	2022	Colón,	*L. (V.) panamensis*
64	B718	2016	Bocas del Toro	*L. (V.) panamensis*
65	B705	2016	Bocas del Toro	*L. (V.) panamensis*
66	L21-05	2021	Darién	*L. (V.) panamensis*
67	L699	2016	Bocas del Toro, Panamá	*L. (V.) panamensis*
68	L22-433	2022	Colón	*L. (V.) panamensis*
69	L22-441	2022	Panamá	*L. (V.) panamensis*
70	L22-443	2022	Coclé	*L. (V.) panamensis*
71	L693	2016	Bocas del Toro	*L. (V.) panamensis*
72	L364	2016	Coclé	*L. (V.) panamensis*
73	L470	2016	Coclé	*L. (V.) panamensis*
74	L454	2016	Coclé	*L. (V.) panamensis*
75	L446	2016	Coclé	*L. (V.) panamensis*
76	L445	2016	Coclé	*L. (V.) panamensis*
77	L697	2016	Bocas del Toro	*L. (V.) panamensis*

78	L527	2016	Coclé	*L. (V.) panamensis*
79	L477	2016	Coclé	*L. (V.) panamensis*
80	L301130003	2022	Panamá Oeste	*L. (V.) panamensis*
81	C22-456	2022	Panamá Oeste	*L. (V.) panamensis*
82	L22-274	2022	Bocas del Toro	*L. (V.) panamensis*
83	L301170004	2022	Colón	*L. (V.) panamensis*
84	L18-166	2018	Panam Oeste	*L. (V.) panamensis*
85	L22-472	2022	Panamá	*L. (V.) panamensis*
86	L18-302	2018	Panama Oeste	*L. (V.) panamensis sp.1*
87	C22-462	2022	Colón	*L. (V.) panamensis*
88	L30201001	2022	Colón	*L. (V.) panamensis*
89	L22-433	2022	Colón	*L. (V.) panamensis*
90	L30131001	2022	Panamá	*L. (V.) panamensis*
91	L01160004	2022	Bocas del Toro	*L. (V.) panamensis*
92	L01250003	2022	Panama Oeste	*L. (V.) panamensis*
93	L22-425	2022	Colón	*L. (V.) panamensis*
94	L22-424	2022	Panamá Oeste	*L. (V.) panamensis*
95	L22-415	2022	Panamá Oeste	*L. (V.) panamensis*
96	L22-467	2022	Panamá	*L. (V.) panamensis*
97	L301200002	2022	Colón	*L. (V.) panamensis*
98	L301170001	2022	Panamá Oeste	*L. (V.) panamensis*
99	L301160006	2022	Panamá Oeste	*L. (V.) panamensis*
100	L301250002	2022	Panamá Oeste	*L. (V.) panamensis*

Isolates positive for LRV-1 are highlighted in red color.
