# Supplementary material for: Epidemiology and Genetic Characterization of Leishmania RNA Virus in Leishmania (Viannia) spp. Isolates from Cutaneous Leishmaniasis Endemic Areas in Panama
Source: Microorganisms. 2024 Jun 27;12(7):1317. doi: 10.3390/microorganisms12071317 (PMC11279101; doi:10.3390/microorganisms12071317)

Supplementary Figure S1. Neighbor-Joining phylogenetic tree of the hsp70 sequences from *L. (Viannia)* constructed with MEGAX software. Sequences from this study are highligthed in yellow and named including the sample ID, the GenBank accession number, the *Leishmania* species and the sequence ID. Reference sequences names include the GenBank accession number the *Leishmania* species and the sequence ID. Bootstrap values above 50% (2000 replicates) are shown above to the node branches. All gapped positions were removed for each sequence pair (pairwise deletion option).

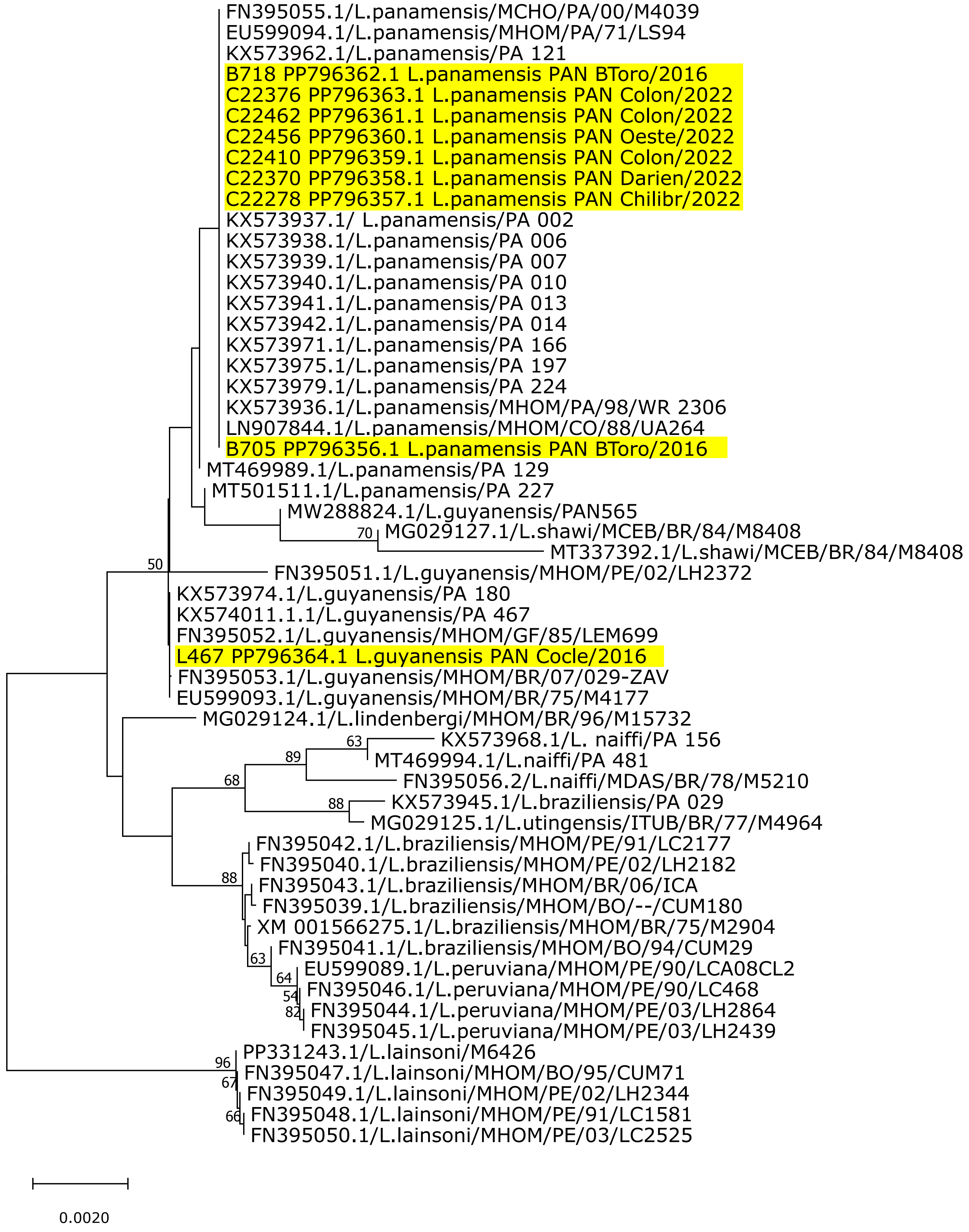

Supplement: Supplementary file 1 [file microorganisms-12-01317-s001.zip › Supplementary Figure S1.pdf]
