# Supplementary material for: Epidemiology and Genetic Characterization of Leishmania RNA Virus in Leishmania (Viannia) spp. Isolates from Cutaneous Leishmaniasis Endemic Areas in Panama
Source: Microorganisms. 2024 Jun 27;12(7):1317. doi: 10.3390/microorganisms12071317 (PMC11279101; doi:10.3390/microorganisms12071317)

Supplementary Figure S2. Bayesian phylogenetic tree of LRV-1 genomes. It was inferred with the concatenated sequences of four genomic fragments representing all three ORFs of the LRV-1 isolate from *L. (V.) guyanensis* in Panama (highlighted in blue) and reference genomes of all genotypes of LRV-1 in *L. (V.) guyanensis* (LVRg). Genotype F (LRV-1 in *L. (V.) braziliensis*, LRVb) is selected as an outgroup to root the tree. Posterior probability >0.6 is reported at clade nodes.

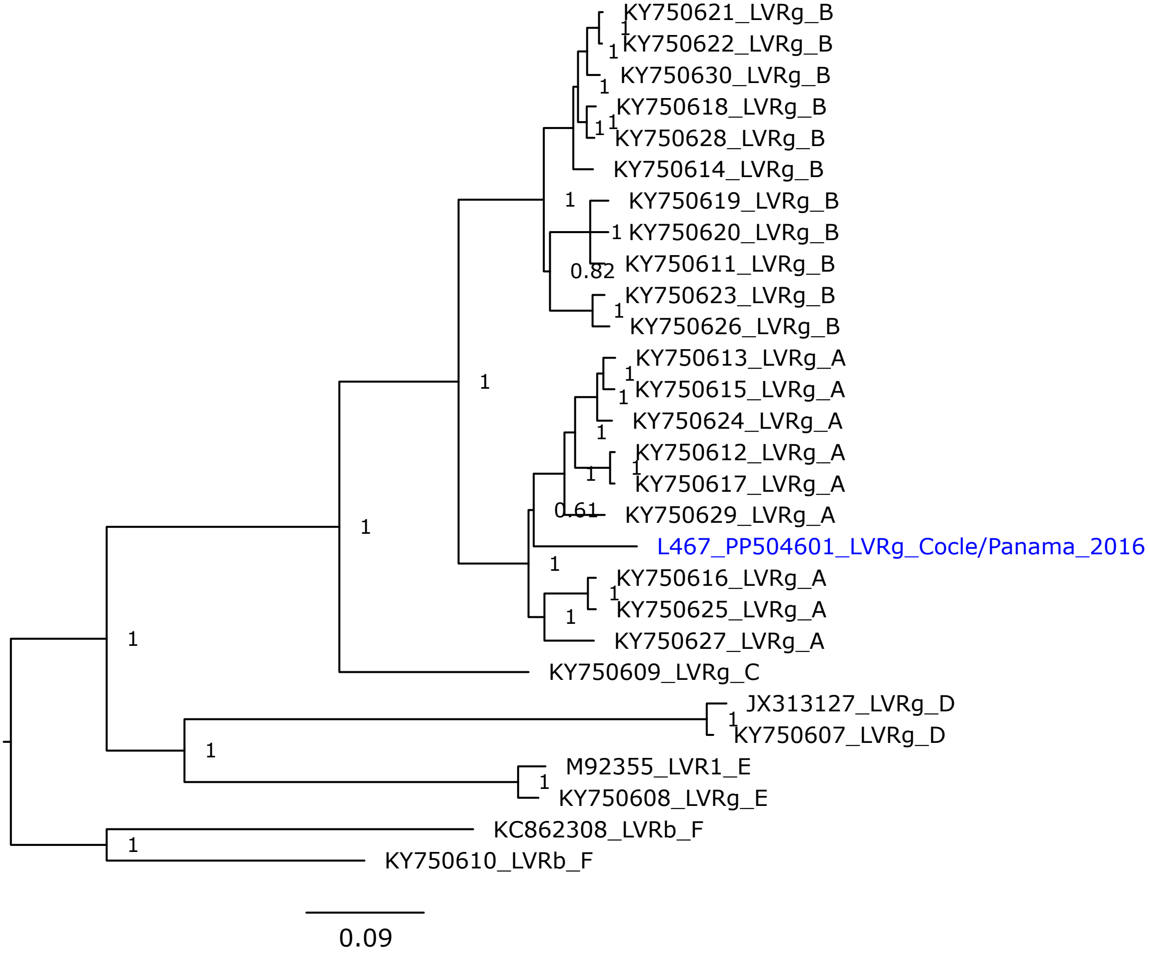

Supplement: Supplementary file 1 [file microorganisms-12-01317-s001.zip › Supplementary Figure S2.pdf]
